# Extensively Drug-Resistant (XDR) and Pandrug-Resistant (PDR) *Acinetobacter baumannii* as Sentinel Indicators of Cumulative System-Level Antimicrobial Pressure in Iraqi Burn and High-Risk Hospital Units

**DOI:** 10.3390/microorganisms14050996

**Published:** 2026-04-29

**Authors:** Sarah Ahmed Hasan, Ali Hasan Mohamed, Gulbahar F. Karim

**Affiliations:** Department of Basic Sciences, College of Nursing, University of Kirkuk, Kirkuk 36001, Iraq

**Keywords:** *Acinetobacter baumannii*, antimicrobial resistance, XDR, PDR, burn units, antimicrobial stewardship

## Abstract

Antimicrobial resistance (AMR) is one of the most significant threats to healthcare systems, particularly in low- and middle-income nations where infection prevention and control, antimicrobial stewardship, and laboratory surveillance might not be optimal. *Acinetobacter baumannii* is a high-risk nosocomial pathogen that has a strong capacity to develop extreme resistance phenotypes. Still, the degree to which extensively drug-resistant (XDR) and pandrug-resistant (PDR) phenotypes reflect the cumulative impact of antimicrobial pressure at unit and system levels in Iraqi hospitals is not fully described. This was a cross-sectional surveillance study that was a laboratory-based investigation done in public hospitals in the Governorate of Kirkuk between January 2024 and January 2025. The BD Phoenix system identified 80 non-duplicate *A. baumannii* isolates that were obtained in high-risk hospital units. The interpretation of antimicrobial susceptibility testing was done according to CLSI guidelines. Internationally recognized definitions were adjusted to local therapeutic availability to classify isolates as XDR or PDR. Unadjusted odds ratios and Fisher’s exact test were used to assess the associations between the PDR phenotype and the chosen clinical or unit-level variables. Among the 80 isolates, 60 (75%) were XDR and 20 (25%) were PDR. Burn units and wound-related infections were disproportionately represented by PDR isolates. There were significant associations between the PDR phenotype and burn unit admission, wound infection, exposure to invasive devices, long hospitalization (greater than 14 days), and previous exposure to broad-spectrum antibiotics. ICU admission and respiratory infection were not significantly related. Cefepime had in vitro activity only in a subset of XDR isolates. Extreme resistance phenotypes can be used as convenient sentinel measures of cumulative antimicrobial pressure and system-level stress in resource-limited environments. There is an urgent need to strengthen infection prevention and control, antimicrobial stewardship, and laboratory surveillance to preserve the remaining therapeutic options.

## 1. Introduction

One of the most urgent challenges to the modern healthcare system is antimicrobial resistance (AMR) due to its effects on the effectiveness of antibiotic therapy, duration of hospital stay, healthcare expenditure, and patient outcomes. It is particularly acute in intensive care units (ICUs) and other high-dependency care facilities, where patients are often subjected to invasive devices, broad-spectrum antibiotics, and prolonged hospitalizations. Low- and middle-income countries are particularly vulnerable due to the lack of diagnostic capacity, suboptimal implementation of antimicrobial stewardship, and weaknesses in the governance of antibiotic use [1,2,3,4,5,6].

*Acinetobacter baumannii* has emerged as a leading problematic healthcare-associated pathogen worldwide. Its resistance to environmental factors, including hospital surfaces, ability to withstand unfavorable environmental conditions, and ability to develop numerous mechanisms to resist antibiotics, especially antibacterial agents, has led to it being a leading cause of ventilator-associated pneumonia, bloodstream infection, and wound infection, especially in ICUs and burn units [1,3,7]. *A. baumannii* infections are linked to high morbidity, mortality, and healthcare costs [2,3,7].

Carbapenem-resistant *A. baumannii* has been designated as a critical-priority pathogen by the World Health Organization [1]. Resistance patterns in this regard cannot be perceived solely as a microbiological phenomenon; they also can be a manifestation of system-level pressures, such as antimicrobial prescribing practices, infection prevention performance, laboratory capacity, and hospital governance [1,3,6,8]. As such, extreme resistance phenotypes could offer insight into the larger structural stress of healthcare systems [3,8].

It is observed that in Iraq, healthcare settings with overcrowding, inadequate isolation facilities, suboptimal infection control practices, and extensive empirical use of broad-spectrum antibiotics can contribute to the emergence and transmission of highly resistant pathogens [4,5]. Though past reports have outlined antimicrobial resistance in Iraqi hospitals, much of the available literature is descriptive, with limited understanding of how clinical exposures interact with broader health-system drivers [4,6].

There is still limited evidence on the role of burn unit admissions, exposure to invasive devices, prolonged hospitalization, and previous antimicrobial exposure in the development of extensively drug-resistant (XDR) and pandrug-resistant (PDR) *A. baumannii* in Iraqi environments [4,5,6,9]. The clustering of these extreme phenotypes in high-risk hospital settings is significant to infection prevention, antimicrobial stewardship, and policy-oriented surveillance [6,8,9].

Consequently, this paper aims to describe XDR and PDR *A. baumannii* isolates that are obtained in high-risk hospital units of public hospitals in Kirkuk governorate and analyze their implications on infection prevention, antimicrobial stewardship, and health policy in a resource-constrained environment.

## 2. Materials and Methods

### 2.1. Design and Setting of the Study

The study was intended to be a cross-sectional laboratory-based surveillance study that was going to be carried out at the public hospitals of Kirkuk governorate, Iraq, during the period between January 2024 and January 2025. Surveillance was targeted to high-risk clinical units such as intensive care units, burn units, surgical wards, medical wards and emergency departments where healthcare-associated infections and antimicrobial resistance are more prone to develop and persist.

This was done as an isolate-based surveillance study as opposed to a denominator-based prevalence study. The expanded surveillance construct was composed of routine clinical samples that were handled in participating hospital laboratories of patients with suspected healthcare-associated infections throughout the study period. In the current analysis, non-duplicate *Acinetobacter baumannii* isolates that were obtained in high-risk hospital units were considered.

Eighty clinical isolates of *A. baumannii* were identified and examined as non-duplicates. The first isolate of a patient was selected only to prevent the overrepresentation of recurrent or persistent isolation from the same person. As such, the study was not aimed at estimating the prevalence of *A. baumannii* in all processed specimens or in all hospitalized patients; the purpose of the study was to describe clinically relevant resistance phenotypes in confirmed isolates of high-risk hospital units.

### 2.2. Context of Healthcare-Associated Infection

The surveillance setting of the current research was limited to the patients under the care of hospital units that are generally linked with the risk of contracting healthcare-associated infections. To use this work, healthcare-associated infection context is defined as infections that are suspected to have been acquired in the context of healthcare delivery, especially in patients who have been exposed to hospitalization, invasive procedures or high-risk care settings, as per conventional infection prevention and control models [9].

### 2.3. Bacterial Identification

Preliminary bacterial identification was carried out by employing conventional microbiological techniques, such as Gram staining, morphology of the colonies, and routine biochemical tests. A presumptive identification of all *Acinetobacter* isolates to the species level with the BD Phoenix automated microbiology system (Becton Dickinson, Franklin Lakes, NJ, USA) was then done to increase standardization and consistency across the participating hospitals.

### 2.4. Antimicrobial Susceptibility Testing

The BD Phoenix automated system was used to carry out antimicrobial susceptibility testing as per the instructions of the manufacturer. This platform has been applied in common clinical microbiology workflows and has been assessed to be useful in susceptibility testing, including the detection of carbapenem resistance [10].

The Clinical and Laboratory Standards Institute (CLSI) criteria and the latest breakpoint updates at the moment of analysis were used to interpret susceptibility results [11]. Since differences between CLSI and European Committee on Antimicrobial Susceptibility Testing (EUCAST) interpretive criteria have been observed, CLSI breakpoints were used consistently across the study to reduce categorical inconsistency and maintain methodological consistency [12].

Ceftazidime, piperacillin-tazobactam, ciprofloxacin, gentamicin, amikacin, imipenem, meropenem, tigecycline, cefepime, colistin, and minocycline were included in the antimicrobial panel. These agents are the main antimicrobial classes that are regularly used in the treatment of *A. baumannii* infections in the local clinical environment.

### 2.5. Phenotyping Resistance Phenotypes and Definitions

The phenotypes of resistance were classified based on the internationally agreed consensus definitions suggested by Magiorakos et al. [13]. The XDR isolates were considered those that were not susceptible to any of the agents except one or two categories of antimicrobial agents.

Operationally, PDR isolates were those isolates that were non-susceptible to all antimicrobial agents within the study panel and included last-line agents like colistin and minocycline within routine clinical practice in the participating hospitals. This mode of operation was to capture clinically significant therapeutic exhaustion on the basis of local resource circumstances and is consistent with the pragmatic understanding of extreme phenotypes of resistance in resource-constrained environments [13].

### 2.6. Data Analysis

The chi-square test or the Fisher exact test, depending on the appropriate test, was used to analyze categorical variables and a *p*-value of less than 0.05 was considered to be statistically significant. Exact methods were favored in the 2 × 2 comparisons that had zero cells. The odds ratios (ORs) were estimated unadjusted where possible; where there was full separation, the ORs were treated as non-estimable (infinite).

Though multivariable logistic regression would have normally been employed to adjust for potential confounders, it was not statistically suitable in this dataset since full separation existed in selected predictors. In particular, all PDR isolates were found in exposed patients in the variables of invasive device use and hospital stay of 14 days or longer, which led to unstable and imprecise regression estimates. Considering this limitation of the structure and the fact that PDR events are relatively few, unadjusted odds ratios and exact tests were deemed the most transparent approach to analysis. Further research based on multicenter studies needs to use penalized logistic regression or exact logistic regression to provide more robust estimations of adjusted associations in sparse data settings.

## 3. Results

### 3.1. Distribution of Acinetobacter baumannii Isolates

Eighty non-duplicate *Acinetobacter baumannii* clinical isolates were obtained in public hospitals in the governorate of Kirkuk, Iraq. Several high-risk hospital units were sampled to obtain isolates. The highest number of isolates was 32 in intensive care units (40.0%), followed by 16 in burn units (20.0%). The most common were surgical wards with 12 isolates (15.0%), medical wards with 10 isolates (12.5%), and emergency departments with 10 isolates (12.5%). Collectively, these units comprised all isolates that were analyzed (*n* = 80).

As far as specimen source is concerned, respiratory samples, such as endotracheal aspirates and sputum, were most likely to yield *A. baumannii*, which is in accordance with the ecology of this pathogen in critically ill and heavily exposed hospitalized patients.

### 3.2. Antimicrobial Resistance Phenotypes

The levels of resistance in the recovered isolates were significantly high. Out of the 80 *A. baumannii* isolates:

- A total of 60 (75.0%) were extensively drug-resistant (XDR) and susceptible to only one or two antimicrobial categories.
- A total of 20 (25.0%) were classified as pandrug-resistant (PDR) and were not susceptible to any of the tested agents, including colistin and minocycline.

In this set of isolates, no completely susceptible isolates were identified and no isolates with conventional multidrug resistance only were observed.

### 3.3. XDR and PDR Isolates Distribution by Hospital Unit

Table 1 presents a summary of the distribution of resistance phenotypes by hospital unit. Burn units had the highest concentration of PDR isolates, with 10 of 16 isolates (62.5%) showing the PDR phenotype. In contrast, although the highest overall number of isolates was obtained from ICUs, the isolates originating from ICUs were mainly XDR and not PDR.

### 3.4. XDR and PDR Isolates Distribution by Clinical Specimen

Table 2 indicates that wound specimens constituted the most common source of PDR isolates, with 12/20 PDR isolates (60.0%) represented by wound specimens. The 6 PDR isolates (30.0%) in respiratory specimens were compared to the remaining isolates from blood, catheter tip, and urine.

### 3.5. Risk Factors Linked to the PDR Phenotype

Comparative pandrug-resistant (PDR) and extensively drug-resistant (XDR) isolates of *A. baumannii* were found to be not randomly distributed but were concentrated in high-risk clinical and unit-level settings (Table 3).

PDR phenotype was closely linked with admission to the burn unit. Burn units recorded 10 of 20 PDR isolates versus 6 of 60 XDR isolates, which showed a strong association (OR = 9.0; Fisher’s exact *p* = 0.001).

The same level of association was found with wound-associated infection. Wound isolates represented 12 out of 20 PDR isolates versus 8 out of 60 XDR isolates (OR = 9.75; Fisher’s exact *p* = 0.001).

In comparison, the PDR phenotype did not have a significant association with ICU admission, even though the number of ICU isolates was high overall (OR = 0.56; *p* > 0.05). PDR was also not significantly related to respiratory infection (OR = 0.64; *p* > 0.05).

It is worth noting that all PDR isolates were observed in patients with exposure to invasive devices, such as mechanical ventilation or intravascular catheterization, while some XDR isolates showed no exposure to devices. The odds ratio for these variables could not be estimated directly due to complete separation, but Fisher’s exact test showed a statistically significant relationship (*p* < 0.01).

This was also observed with prolonged hospitalization. PDR isolates were all obtained from patients who had a hospital stay of 14 days or more, while half of the XDR isolates were linked to this exposure. This again made the odds ratio non-estimable, but a significant exact-test association was observed (*p* < 0.001).

A history of previous exposure to broad-spectrum antibiotics was also closely linked to the PDR phenotype. PDR isolates were associated with prior exposure in 19 of 20 cases, versus 34 of 60 XDR isolates, which resulted in a significant association (OR = 14.53; *p* < 0.01).

Collectively, these results indicate that burn unit admission, wound infection, exposure to invasive devices, length of stay, and prior exposure to broad-spectrum antimicrobials have strong associations with the development of pandrug-resistant *A. baumannii* and are aligned with cumulative clinical and system-level selective pressures.

### 3.6. Therapeutic Implications

The only antimicrobial agent that showed measurable in vitro activity against a subset of XDR *A. baumannii* isolates was cefepime. This observation must be interpreted with caution and should not be considered a direct treatment recommendation unless supported by pharmacokinetic/pharmacodynamic optimization and clinical outcome data. The presence of PDR isolates in a quarter of all isolates, particularly in burn cases, reflects an extremely limited therapeutic landscape and highlights the constrained treatment options for the most vulnerable hospitalized patients. These findings support prioritizing prevention, antimicrobial stewardship, and adjunctive non-antibiotic strategies rather than reliance on increasingly ineffective antimicrobial agents.

## 4. Discussion

This article demonstrates that the burden of extreme antimicrobial resistance in *Acinetobacter baumannii* recovered isolates is substantially high in high-risk hospital units of the state-owned hospitals of Kirkuk governorate in Iraq. The absence of isolates that are entirely susceptible and the presence of XDR and PDR phenotypes indicate a very limited therapeutic landscape, and not a microbiological anomaly within the isolate. These findings are consistent with previous reports that show that the emergence of advanced resistance in *A. baumannii* is more likely to occur in an environment with a high frequency of antimicrobial exposure, longer hospitalization, administration of invasive care, and inadequate infection prevention capacity [14,15].

Absence of traditionally MDR-only isolates cannot be considered definitive evidence that such phenotypes are not present in the broader hospital ecosystem. Instead, it is more likely to be explained by the surveillance design of the study, which selectively targeted high-risk units in which antimicrobial pressure, invasive procedures, and referral of severe cases are concentrated. In such environments, there is a high likelihood of recovery of advanced resistance phenotypes over less resistant strains. Therefore, the existing dataset does not represent the whole range of resistance of *A. baumannii* within the hospital but only in high-risk settings.

Disproportionate representation of PDR isolates in burn units, as well as wound-related infections, has been the most notable observation. Burn patients are particularly vulnerable because they spend considerable time in the hospital, undergo numerous procedures, are exposed to invasive equipment, are on antimicrobial treatment, and the integrity of the skin barrier is constantly compromised. Open wounds may also be a source of environmental contamination, chronic colonization, and cross-transmission. Similar findings have been recorded in previous studies in burn and critical care units, where exposure to burn units was significantly linked to the emergence or persistence of highly resistant *A. baumannii* [16].

The significant correlations observed between the PDR phenotype and burn unit admission, wound infection, invasive device exposure, length of stay, and history of prior use of broad-spectrum antibiotics favor the conclusion that cumulative rather than single isolated selective pressures are more suitable to explain the existence of pandrug resistance. PDR, on the other hand, did not show any significant association with ICU admission or respiratory infection in this data set, suggesting that extreme resistance might be driven by the interaction of repeated exposure patterns and unit-specific selective environments and not just critical care admission [15,16,17].

The fact that even cefepime was still noted to be active in vitro in only some of the XDR isolates also illustrates how limited treatment choices are becoming. This observation must, however, be considered with caution because in vitro susceptibility does not necessarily translate into clinical efficacy, particularly in patients with critical illness, altered pharmacokinetics, altered tissue penetration or severe underlying disease [18]. The reduced profile of residual activity shown here, however, points out the practical fragility of the remaining therapeutic options.

These findings, systematically, support the perception that extreme resistance phenotypes may be used as warning signs of structural stress in healthcare provision. Such stresses may include the cumulative use of antimicrobials, overuse of devices, overcrowding, delayed microbiological response, environmental contamination, and infection prevention lapses [19,20]. The aim of the current research was not to produce national prevalence estimates and the findings cannot be deemed nationally representative. Instead, they give a sentinel picture of resistance mechanisms in one governorate of publicly funded hospitals in resource-limited circumstances. They are more generalizable in regard to conceptual transferability to other high-risk hospital contexts in Iraq and other low-resource conditions.

Overall, the paper suggests that high-risk hospital units should be equipped with better infection prevention and control, antimicrobial stewardship, and laboratory-based surveillance. Such interventions are more practicable in the short term instead of depending on therapeutic rescue interventions that are becoming increasingly constrained [19,20].

### 4.1. Health Policy Implications

The prevalence of XDR and PDR *Acinetobacter baumannii* among high-risk hospital units underscores the need to strengthen antimicrobial stewardship and infection control within Iraq and, in particular, in the burn units and other high-risk settings that have the highest cumulative antimicrobial pressures and risk of transmission [19,20]. The priority areas include giving more focus on the use of last-line antibiotics, audit-and-feedback, and enhancing processes of evidence-based prescribing, which could reduce selective pressure in resource-limited settings [17,19].

Localization of PDR cases within burn and wound cases also supports the need to intensify infection prevention and control efforts. These include hand hygiene, environmental decontamination, isolation or cohorting of patients where feasible, and continuous monitoring of the unit. In addition, reducing unnecessary invasive device use, incorporation of device-related infection prevention bundles, as well as promotion of antimicrobial de-escalation based on microbiological findings, are also likely to decrease exposure-related selective pressure [16,18,20].

There is also the need to increase laboratory capacity and integrate AMR data into coordinated hospital and national surveillance systems to enable timely detection and response [19,20,21,22]. This work was not aimed at establishing a national threshold, but the results are in favor of a pragmatic early warning system. Local review of PDR isolates in high-risk units should be done after their identification, especially in the case of recurrent identification. Likewise, a PDR percentage of over 10 percent of *A. baumannii* isolates in burn units or other units with high-risk situations can be considered as a tentative alert limit to strengthen infection control, stewardship review, environmental evaluation as well as report escalation. This is a hypothesis-generating threshold that will need to be prospectively tested in multicenter surveillance systems in Iraq before adoption of policies. Besides that, nanoemulsions, medicinal plants, and other novel approaches may offer supportive alternative strategies for the management of PDR infections in settings where conventional treatment options are limited [23,24,25].

### 4.2. Limitations and Perspectives

This study has a number of limitations. First, it was not a prevalence study, but it was an isolate-based laboratory surveillance study; hence, it does not give incidence or prevalence estimates based on all processed clinical specimens. Second, not all included isolates had fully linked demographic data, which limited age- and sex-specific subgroup analyses. Third, the research was carried out in public hospitals within Kirkuk governorate, although the sample size was too small to provide a robust hospital-level comparative analysis of the PDR proportions.

Fourth, the study was based on phenotypic antimicrobial susceptibility testing and was not accompanied by patient-level clinical outcome variables such as mortality, severity of illness, therapeutic response, and standardized distinction between infection and colonization. As such, the study may be viewed as more of a microbiological and unit-level surveillance study as opposed to a clinical cohort outcome study.

The other shortcoming is the absence of molecular characterization. No PCR-based resistance tests were done to identify some of the most important resistance determinants, such as OXA-type carbapenemases, NDM, VIM, or other resistance-related genes. In the same way, molecular mechanisms of efflux pumps, aminoglycoside resistance determinants, and fluoroquinolone resistance mechanisms were not investigated. In this way, it was not possible to determine the genetic architecture of the observed phenotypes.

Additionally, no clonality testing was conducted, such as multilocus sequence typing (MLST), and no distribution of minimal inhibitory concentration (MIC) was done in a comprehensive quantitative format. Phenotypic susceptibility information combined with molecular resistance profiling and clonality analysis, together with more detailed MIC distributions, should be used in future multicenter studies to differentiate between local emergence and transmission, provide better epidemiological meaning, and enhance policy relevance.

Lastly, the study was limited to a single governorate, and thus, the results cannot be considered nationally representative. Nevertheless, the research offers a clinically applicable sentinel representation of the extreme resistance phenotypes in the high-risk hospital units in a resource-constrained environment.

## 5. Conclusions

The present study has revealed a substantial burden of extreme antimicrobial resistance in *Acinetobacter baumannii* isolates collected in high-risk units in public hospitals in Kirkuk governorate in Iraq, with a high percentage of isolates showing XDR and PDR phenotypes. These phenotypes, concentrated in burn units and other high-risk environments, indicate that the most vulnerable hospitalized patients are subjected to an environment with significantly limited therapeutic choices.

These observations indicate that the problem of antimicrobial resistance in this context is not just a microbiological phenomenon, but it is also a manifestation of overall health-system pressures, such as antimicrobial use practices, exposure to invasive care, and failures in infection prevention and control. Enhancing antimicrobial stewardship, infection control, and laboratory surveillance must thus be prioritized to avoid further deterioration of effective treatment options.

Though the study was carried out in one governorate and is not nationally representative, it offers a feasible sentinel perspective into resistance dynamics in high-risk, resource-constrained hospital environments. System-level interventions, such as governance fortification, antimicrobial stewardship, and improved surveillance, are needed to mitigate the impact of extreme resistance and enable sustainable healthcare provision.

## Figures and Tables

**Table 1:** Distribution of XDR and PDR *Acinetobacter baumannii* isolates across high-risk hospital units (*n* = 80).

Hospital Unit	Total Isolates (*n*)	XDR (*n*)	PDR (*n*)
**Intensive Care Unit (ICU)**	32	26	6
**Burn Unit**	16	6	10
**Surgical Wards**	12	12	0
**Medical Wards**	10	10	0
**Emergency Department**	10	6	4
**Total**	**80**	**60**	**20**

**Table 2:** Distribution of XDR and PDR *Acinetobacter baumannii* isolates by clinical specimen source (*n* = 80).

Specimen Type	Total Isolates (*n*)	XDR (*n*)	PDR (*n*)
**Respiratory (endotracheal aspirate/sputum)**	30	24	6
**Wound Swab**	20	8	12
**Blood**	12	12	0
**Catheter Tip**	10	10	0
**Urine**	8	6	2
**Total**	**80**	**60**	**20**

**Table 3:** Clinical and unit-level risk factors associated with PDR *Acinetobacter baumannii* compared with XDR isolates.

Risk Factor	XDR (*n* = 60)	PDR (*n* = 20)	Odds Ratio (OR)	Interpretation
**Burn Unit Admission**	6	10	9.0	Strong association with PDR phenotype
**ICU Admission**	26	6	0.56	Not statistically associated
**Wound Infection**	8	12	9.75	Strong association with PDR phenotype
**Respiratory Infection**	24	6	0.64	Not statistically significant
**Use of Invasive Devices**	40	20	Not estimable	100% of PDR exposed
**Hospital Stay ≥ 14 Days**	30	20	Not estimable (∞)	All PDR had prolonged stay
**Prior Broad-Spectrum Antibiotics**	34	19	14.53	Strong association

Odds ratios are unadjusted. In variables with zero cells, ORs were not estimable; statistical significance was assessed using Fisher’s exact test.

## Data Availability

The datasets generated and analyzed during the current study are available in the Figshare repository at https://doi.org/10.6084/m9.figshare.31111786.
